# Antioxidative and Anti-Melanogenic Activities of Bamboo Stems (*Phyllostachys nigra* variety henosis) via PKA/CREB-Mediated MITF Downregulation in B16F10 Melanoma Cells

**DOI:** 10.3390/ijms19020409

**Published:** 2018-01-30

**Authors:** Moon-Hee Choi, Han-Gyo Jo, Ji Hye Yang, Sung Hwan Ki, Hyun-Jae Shin

**Affiliations:** 1 Department of Chemical Engineering, Graduate School of Chosun University, Gwangju 61452, Korea; aamoony1222@naver.com (M.-H.C.); biochem2446@naver.com (H.-G.J.); 2 College of Pharmacy, Chosun University, Gwangju 61452, Korea; uranus2k@nate.com (J.H.Y.); shki@chosun.ac.kr (S.H.K.)

**Keywords:** bamboo stem, *Phyllostachys nigra* var. henosis, anti-melanogenesis, antioxidation, B16F10 melanoma cells, whitening cosmetics

## Abstract

*Phyllostachys nigra* var. henosis, a domestic bamboo species, has been attracting much attention; its bioactive compounds (especially in the leaf) show antioxidant, anti-inflammatory, and anti-obesity activities. Little information is available on the antioxidative and anti-melanogenetic activities of the bioactive compounds in bamboo stems. The anti-melanogenic and antioxidative activities of the EtOAc fraction (PN3) of a *P. nigra* stem extract were investigated in a cell-free system and in B16F10 melanoma cells. PN3 consisted of a mixture of flavonoids, such as catechin, chlorogenic acid, caffeic acid, and *p*-coumaric acid. The antioxidant activity (2,2-diphenyl-1-picrylhydrazyl (DPPH), 2,2′-azino-bis(3-ethylbenzothiazoline-6-sulfonic acid (ABTS)), and hydroxyl radical scavenging) was evaluated, as well as the inhibition of reactive oxygen species (ROS) produced by the Fenton reaction. PN3 showed in vitro tyrosinase inhibition activity with the half maximal inbihitory concentration (IC_50_) values of 240 μg/mL, and in vivo cytotoxic concentration ranges > 100 μg/mL. The protein expression levels and mRNA transcription levels of *TYR*, *TRP-1*, and *MITF* were decreased in a dose-dependent manner by the treatment with PN3. PN3 interfered with the phosphorylation of intracellular protein kinase A (PKA)/cAMP response element-binding protein (CREB), demonstrating potent anti-melanogenic effects. PN3 could inhibit PKA/CREB and the subsequent degradation of microphthalmia-associated transcription factor (MITF), resulting in the suppression of melanogenic enzymes and melanin production, probably because of the presence of flavonoid compounds. These properties make it a candidate as an additive to whitening cosmetics.

## 1. Introduction

Melanin, composed of pheomelanin and eumelanin, is synthesized in the melanosomes of melanocytes [1]. Synthesized melanin is located in the skin, hair, and eyes, determines skin color and protects from UV damage. The abnormal synthesis of melanin causes skin conditions such as albinism, melasma, freckles, and post-inflammatory hyperpigmentation. In the first two steps of melanin biosynthesis, tyrosinase (EC 1.14.18.1) is the key enzyme in catalyzing the hydroxylation of L-tyrosine to 3,4-dihydroxyphenylalanine (DOPA) and the oxidation of DOPA to dopaquinone. Thus, the inhibition of tyrosinase activity may be considered as an important role in the development of whitening cosmetics. Recently, many researchers have been trying to develop new whitening cosmetics with natural compounds including phytochemicals, such as polyphenol, flavonoids, and carotenoids. Polyphenols are one of the strongest candidates for use, among many plant-derived whitening materials.

Bamboo is a large woody grass belonging to the family Poaceae, which consists of useful plants containing various polyphenol components. In a previous study on bamboo stems, it was reported that its consumption decreased serum total cholesterol and low-density lipoprotein (LDL) levels in women [2]. Bamboo shoot oil extracted from *Phyllostachys edulis* was documented to decrease total cholesterol and LDL, as well as the levels of serum triacylglycerol, phytosterol, and lipoprotein lipase. Moreover, bamboo stem extracts ameliorated fatty liver disease and increased the cholesterol content in the feces of rats treated with a high-fat diet. These lipid-lowering effects were attributed to the inhibition of cholesterol absorption [3]. Furthermore, the acetylcholine content is high in bamboo stems; acetylcholine is an important neurotransmitter in the cholinergic nervous system of vertebrates and insects [4]. In the last few years, studies on the bamboo stem have been mostly focused on its antioxidant, cholesterol-lowering, and fat-reducing abilities. To our knowledge, however, there are no reports on the effect of bamboo stem extracts containing many flavonoids with respect to both antioxidant and anti-melanogenesis activities. Therefore, this study identifies the major compound and mechanism of the anti-melanogenesis effect of a bamboo stem extract.

## 2. Results

### 2.1. Bamboo Stem Extraction and Antioxidative Activities of the Extract

**Figure 1:**
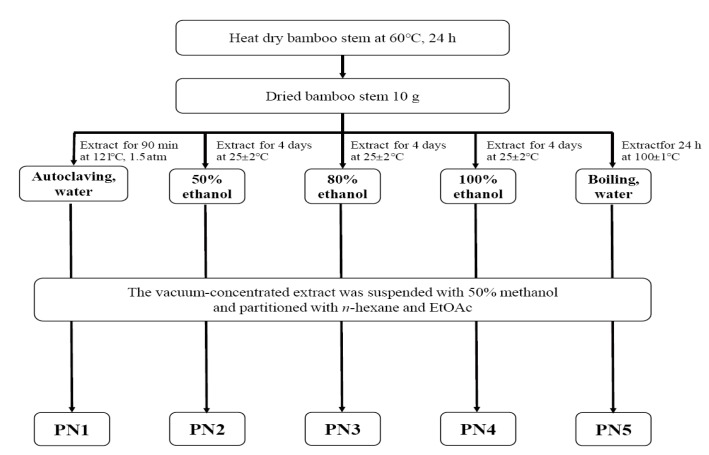
Schematic diagram of the extract preparation from *Phyllostachys nigra* var. henonis.

Domestic bamboo stems (*Phyllostachys nigra* var. henosis) were extracted using water and ethanol (EtOH), and the extracts were then separated into hydrophilic and hydrophobic fractions with methanol (MeOH), *n*-hexane, and ethyl acetate (Figure 1).

The antioxidant properties of bamboo stem extracts were assayed for radical scavenging activities using 2,2-diphenyl-1-picrylhydrazine (DPPH), 2,2′-azino-bis(3-ethylbenzothiazoline-6-sulfonic acid) (ABTS), and hydroxyl radicals (Table 1 and Table S1). Ascorbic acid was used as a positive control. The concentrations of the extracts were from 100 to 5000 μg/mL and the IC_50_ of the ethyl acetate fraction of the 80% EtOH extract (PN3) was 565.63 ± 17.75 μg/mL in the DPPH test, 414.61 ± 35.12 μg/mL in the ABTS test, and 509.17 ± 33.76 μg/mL in the hydroxyl radical test. PN3 had the highest antioxidant activity among all fractions in all radical scavenging test. For PN4, the IC_50_ in ABTS and hydroxyl radical tests was 669.68 ± 35.62 μg/mL and 766.73 ± 22.23 μg/mL, respectively. Ascorbic acid, used as the positive control, showed an IC_50_ of 23.02 ± 0.39 μg/mL in the DPPH test and 51.86 ± 0.72 μg/mL in the ABTS test. In the hydroxyl radical test, the IC_50_ of ascorbic acid was 90.57 ± 0.60 μg/mL. The total polyphenol and total flavonoid contents were determined using photocolorimetric methods. *p*-Coumaric acid (COA) and quercetin (Q) were used as equivalent materials. PN3 presented the highest content of phenolic compounds (Table 2). The total polyphenol and total flavonoid contents of PN3 were 0.179 ± 0.022 *p*-coumaric acid equivalent (COAE) mg/g and 9.57 ± 0.37 quercetin equivalent (QE) mg/g, respectively.

### 2.2. Chemical Composition of the EtOAc fraction of 80% EtOH extract (PN3)

Like other bamboo extracts previously reported [5,6], PN3 is considered to have many flavonoids showing antioxidant activities. Seven flavonoid standards (catechin, chlorogenic acid, caffeic acid, *p*-coumaric acid, ferulic acid, rutin, and luteolin) were chosen to identify the components in PN3. The seven flavonoids in PN3 were analyzed, and their contents were measured (Figure S1A,B). The major component was catechin (701.39 μg/g), followed by chlorogenic acid (297.65 μg/g), and caffeic acid (241.94 μg/g). Also, the *p*-coumaric acid content was 193.11 μg/g in PN3. To confirm the presence of the compounds, liquid chromatography combined with mass spectrometry was applied to confirm the results from the high-performance liquid chromatography (HPLC) analysis (Figure S1C). In the liquid chromatography coupled with quadrupole-time-of-flight mass spectrometry (LC–Q–TOF-MS) analysis, the tentative identification of seven components in PN3 was performed based on the accurate mass data of the elemental composition. The separated main components in PN3 were identified, and their calculated *m*/*z* values and formulas were summarized (Figure S1D). Peak one (retention time (RT) = 5.102 min, *m*/*z* 290.079), peak two (RT = 4.935, *m*/*z* 354.095), peak three (RT = 5.480 min, *m*/*z* 180.042), peak four (RT = 7.323 min, *m*/*z* 164.047), peak five (RT = 8.346 min, *m*/*z* 194.057), peak six (RT = 9.324 min, *m*/*z* 610.153), and peak seven (RT = 12.406 min, *m*/*z* 286.047) had no identical (M + H)^+^ ions. This indicates that all seven peaks were caused by different chemicals. Peaks one, two, and seven were identified as catechin (C_15_H_14_O_6_), chlorogenic acid (C_16_H_18_O_9_), and luteolin (C_15_H_10_O_6_), respectively. Caffeic acid (peak three; C_9_H_8_O_4_), *p*-coumaric acid (peak four; C_9_H_8_O_3_), and ferulic acid (peak five, C_10_H_10_O_4_) were also observed in PN3. Peak six, which was identified as rutin (C_27_H_30_O_16_), had the largest molecular weight among the seven components.

### 2.3. Inhibition of Fenton’s Reaction

**Figure 2:**
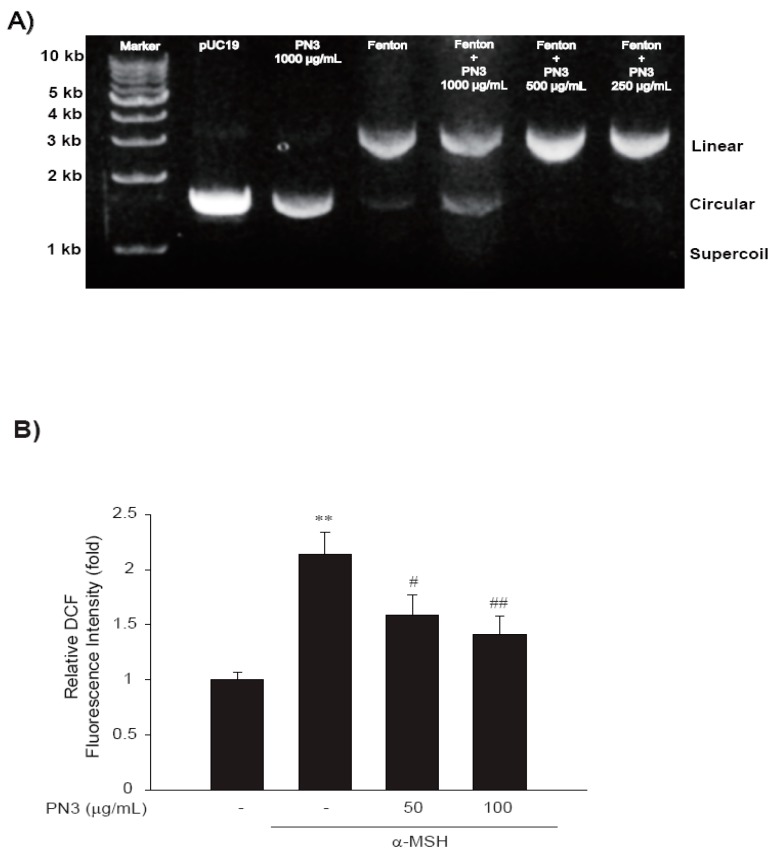
(**A**) DNA protection assay of PN3. Treatment of pUC19 plasmid with the hydroalcoholic extract in the absence and presence of the DNA nicking/damage-causing Fenton’s reagent, followed by a densitometric analysis of the relaxed, linear, and circular forms of pUC19 DNA (on agarose gel). (**B**) Reactive oxygen species (ROS) induced in B16F10 cells at various concentrations of 3% H_2_O_2_ and α-melanocyte-stimulating hormone (α-MSH) (1 μM), treated after 1 h of exposure. Reactive oxygen species induced by α-MSH (1 μM) in B16F10 cells were revealed by staining with 10 mM 2’,7’-dichlorodihydrofluorescein diacetate (DCFH-DA) for 30 min at 37 °C. H_2_O_2_ generation was determined by fluorescence microplate reader. Represent the data of 3 separate experiments (significant as compared with vehicle-treated control, ** *p* < 0.01; significant as compared with α-MSH, # *p* < 0.05, ## *p* < 0.01, bars indicate S.D.).

This assay was based on the ability of extracts to protect the pUC19 plasmid DNA against damage caused by hydroxyl (∙OH) radicals. PN3 showed the inhibition of DNA damage caused by hydroxyl radicals (Figure 2A). The first lane was the pUC19 control, the second lane was pUC19 + PN3, the third lane was Fenton’s reagent + pUC19, and the fourth lane was Fenton’s reagent + pUC19 + PN3 (1000 μg/mL). The Fenton’s reagent group showed one band of circular plasmid, but the PN3 treatment group showed both circular and linear bands. Figure 2A showed the ability of the extracts to reduce Fe^3+^-dependent plasmid DNA nicking. Additionally, α-melanocyte-stimulating hormone (α-MSH) induced reactive oxygen species (ROS) generation in B16F10 cells using a sensitive fluorescent assay method with dichloro-dihydro-fluorescein diacetate (DCFH-DA), whereas the PN3 pretreatment significantly prevented ROS generation by α-MSH (Figure 2B).

### 2.4. Inhibition of In Vitro Tyrosinase Activity and Melanin Synthesis

**Figure 3:**
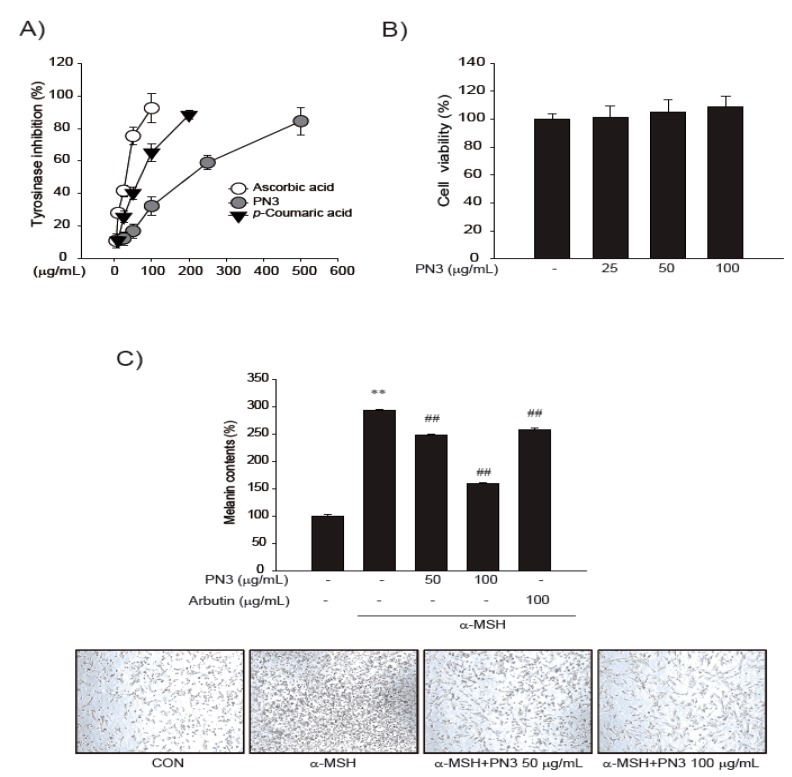
(**A**) The inhibitory effect of mushroom tyrosinase from PN3, ascorbic acid, and *p*-coumaric acid (COA) using a cell-free system; (**B**) measurement of the cytotoxicity of PN3 at 25–100 μg/mL in B16F10 melanoma cells; (**C**) effects of PN3 on melanogenesis in B16F10 cells and morphology changes. B16F10 melanoma cells were cultured for 48 h in the presence of 50–100 μg/mL of PN3 and 100 μg/mL of arbutin as a positive control, or 1 μg/mL of α-MSH. CON: control B16F10 cell line without any treatment. Represent the data of 3 separate experiments (significant as compared with vehicle-treated control, ** *p* < 0.01; significant as compared with α-MSH, ## *p* < 0.01, bars indicate S.D.).

Tyrosinase inhibitory activity was assayed in a cell-free system for primary screening of PN3. An in vitro cell system assay was not performed because we focused on the melanin content in the cell line. The IC_50_ values of PN3, ascorbic acid, and *p*-coumaric acid for tyrosinase inhibition were 243.7, 38.5, and 87.0 μg/mL, respectively (Figure 3A). Cytotoxic concentrations up to 100 μg/mL after 48 h treatment in PN3 had no toxicity (Figure 3B). In Figure 3C, after 48 h of treatment with PN3 (50–100 μg/mL) and α-MSH (1 μg/mL), the melanin content decreased in a dose-dependent manner.

### 2.5. PN3 Downregulated Melanin Synthesis Genes

**Figure 4:**
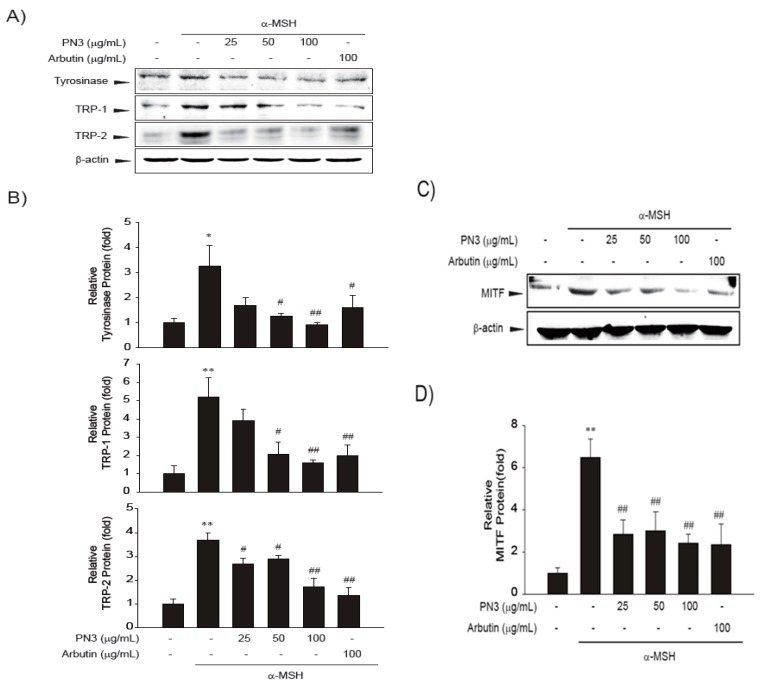
(**A**) Effect of the PN3 extract on the protein expression levels of tyrosinase (TYR), tyrosinase related protein 1 (TRP-1), and tyrosinase-related protein 2 (TRP-2) in B16F10 melanoma cells. B16F10 melanoma cells were treated with the indicated concentrations (25, 50, and 100 μg/mL) of the PN3 extract or with arbutin prior to α-melanocyte-stimulating hormone (α-MSH) treatment for 48 h. The loading control was assessed using a β-actin antibody; (**B**) quantitative analysis of tyrosinase, TRP-1, TRP-2 by western blotting. Cell lysates were subjected to western blotting using antibodies against tyrosinase, TRP-1, and TRP-2; (**C**) effect of the PN3 extract on microphthalmia-associated transcription factor (MITF) protein expression in B16F10 melanoma cells. B16F10 melanoma cells were treated with the indicated concentrations (25, 50, and 100 μg/mL) of the PN3 extract or with arbutin prior to α-MSH treatment for 6 h; (**D**) quantitative analysis of MITF by western blotting. The values in the same column not sharing a common superscript are significantly different by Duncan’s multiple range test (*p* < 0.05). Represent the data of 3 separate experiments (significant as compared with vehicle-treated control, * *p* < 0.05, ** *p* < 0.01; significant as compared with α-MSH, # *p* < 0.05, ## *p* < 0.01, bars indicate S.D.).

Melanogenesis mainly depends on the regulation of melanogenic proteins such as tyrosinase, tyrosinase-related protein 1 (TRP-1), and tyrosinase-related protein 2 (TRP-2). A western blot analysis was performed to determine whether the inhibitory effects of PN3 were related to the regulation of melanogenesis-related proteins. As shown in Figure 4A,B, the protein expression levels of tyrosinase, TRP-1, and TRP-2 were increased by treatment with α-MSH, while PN3 led to a significant decrease in tyrosinase, TRP-1, and TRP-2 in B16F10 cells. Moreover, α-MSH-induced (1 μg/mL) microphthalmia-associated transcription factor (MITF) expression was also decreased in a dose-dependent manner by treatment with PN3 at 25–100 μg/mL (Figure 4C,D).

**Figure 5:**
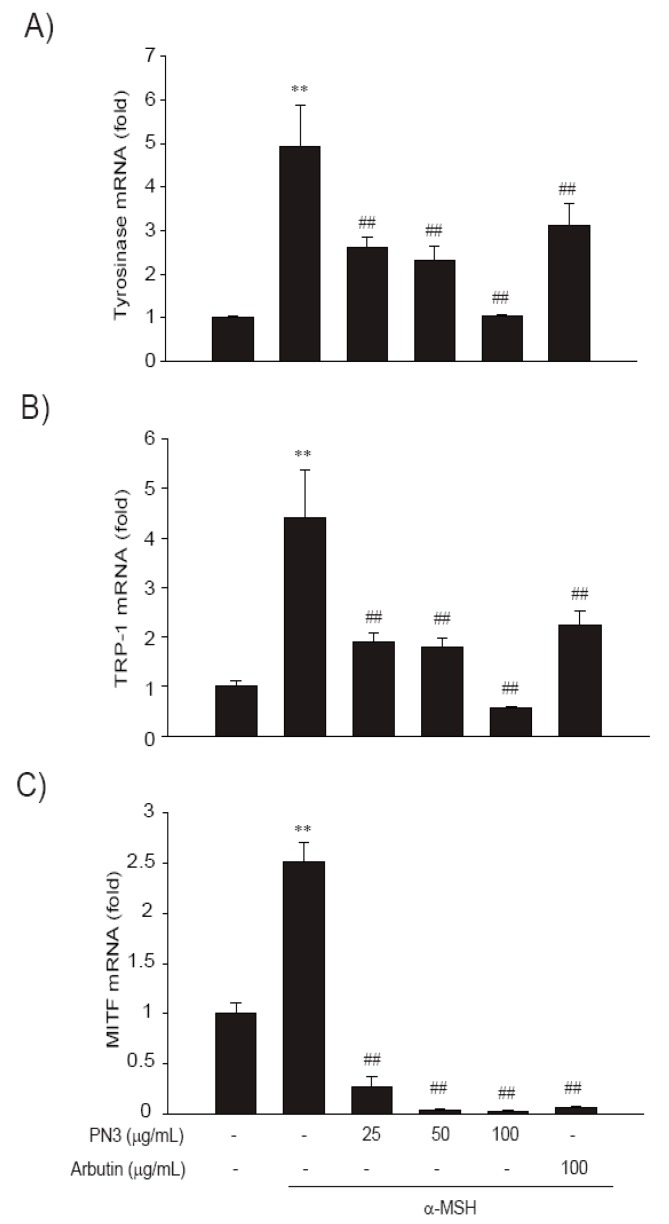
(**A**) Effect of PN3 on tyrosinase (*TYR*), (**B**) tyrosinase-related protein 1 (*TRP*-1), and (**C**) microphthalmia-associated transcription factor (*MITF*) mRNA levels in B16F10 cells. Total cellular proteins (20 μg/lane) were subjected to 10% sodium dodecyl sulfate polyacrylamide gel electrophoresis (SDS–PAGE). The relative intensities of *TYR*, *TRP*-1, and *MITF* mRNA compared with that of *SYBR* Green were determined using Quantity One software (BioRad, Hercules, CA, USA). Represent the data of 3 separate experiments (significant as compared with vehicle-treated control, ** *p* < 0.01; significant as compared with α-MSH, ## *p*< 0.01, bars indicate S.D.).

**Figure 6:**
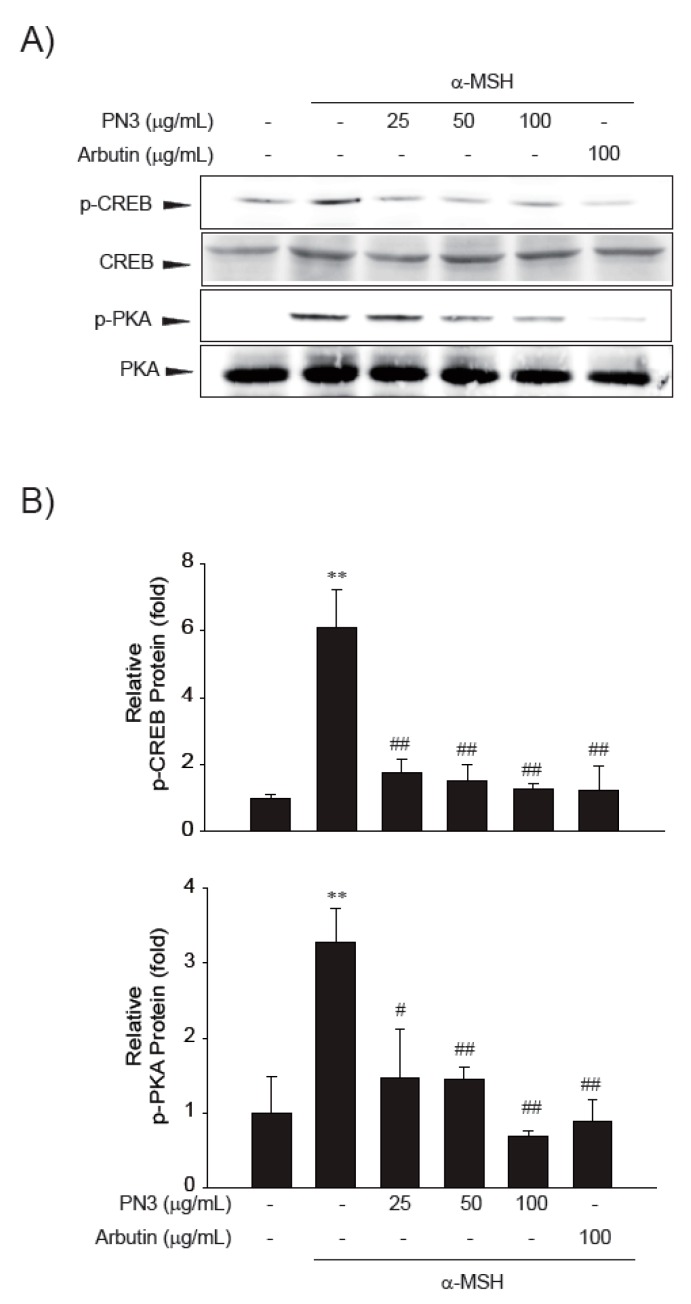
(**A**) Effects of the PN3 extract and of arbutin on the phosphorylation of cyclic adenosine monophosphate (cAMP) response element-binding protein (p-CREB) and of protein kinase A (p-PKA), and on CREB and PKA protein expression in B16F10 melanoma cells. B16F10 melanoma cells were treated with the indicated concentrations (25, 50, and 100 μg/mL) of the PN3 extract or with arbutin prior to α-melanocyte-stimulating hormone (α-MSH) treatment for 3 h; (**B**) quantitative analysis of p-CREB, CREB, p-PKA, and PKA by western blotting. The values in the same column not sharing a common superscript are significantly different by Duncan’s multiple range test (*p* < 0.05). Represent the data of 3 separate experiments (significant as compared with vehicle-treated control, ** *p* < 0.01; significant as compared with α-MSH, # *p* < 0.05, ## *p* < 0.01, bars indicate S.D.).

Next, the effect of the PN3 extract on the expression of melanin production-related gene was assessed by mRNA expression analysis (Figure 5). The mRNA expression of all genes (*TYR*, *TRP*-1, and *MITF*) was triggered by α-MSH, whereas PN3 treatment showed a significantly decreased mRNA expression (25–100 μg/mL). Especially, 100 μg/mL of PN3 was more effective in decreasing mRNA expression than other concentrations. Moreover, PN3 showed a more potent inhibitory activity than the positive control, arbutin. Melanin formation and melanogenic gene expression is regulated by various signaling pathways, including, primarily MITF signaling as well as protein kinase A (PKA), cyclic adenosine monophosphate (cAMP) response element-binding protein (CREB), mitogen-activated protein kinases (MAPKs), phosphatidylinositol-4,5-bisphosphate 3-kinase (PI3K), protein kinase B (AKT), and glycogen synthase kinase 3 (GSK-3) [7,8,9,10]. To elucidate the mechanism underlying the melanogenic effect of PN3, B16F10 cells were exposed to PN3 (25–100 μg/mL) for the indicated time periods, and the protein extracts were then analyzed by western blotting analysis. As shown in Figure 6, PN3 preincubation inhibited the α-MSH-induced phosphorylation of PKA/CREB. These results suggested that the suppressive mechanism of PN3 was related to the inhibition of PKA/CREB signaling.

## 3. Discussion

As a result of the various pathways and related factors involved in melanogenesis, researchers have found many compounds in plants with anti-melanogenesis effects. Especially, reactive oxygen species caused by ultraviolet, a major factor of melanogenesis, promote melanin pigment formation in skin cells. Scavenging the radicals and ROS was significantly effective in inhibiting the formation of melanin [11]. Polyphenols, including flavonoids, which have antioxidant activities, can have an inhibitory effect on melanogenesis by removing ROS. *Phyllostachys pubescens* stem was extracted with solvent and fractionated several times with *n*-butanol and chloroform [12]. In the DPPH assay, the chloroform and MeOH (10:1–5:1) fractions showed powerful antioxidant activity; the active compound was identified as *p*-coumaric acid, which is a phenolic compound. *Phyllostachys bambusoides* stem was extracted with EtOH [13], and the DPPH and superoxide dismutase (SOD) activity of the extract were measured. The IC_50_ values of the extract and of β-carotene (as a positive control) were 116.75 μg/mL and 18.02 μg/mL, respectively. The extract showed 43.88% SOD activity at 0.92 mg/mL, while the positive control (ascorbic acid) showed 76.06% SOD activity at 0.184 mg/mL. The biological activities and phytochemicals of all parts of bamboo (*P. pubescens*) were assayed [14]. The EtOH extracts of the inner culm had a strong antioxidant activity, with an oxygen radical absorbance capacity (ORAC) value of 1.35 mg TE/mg. Regarding the SOD-like activity, the EtOH extracts of the inner culm and outer culm showed high antioxidant activities (0.1–1.0 U/μg). The EtOH extract of the inner culm had the strongest ABTS radical scavenging activity among all extracts (IC_50_ = 88.5 μg/mL). Concerning the results of this study, a significant antioxidant activity was detected in the EtOH extract of the bamboo stem. Other studies have reported similar antioxidant activities as those reported in this study. The differences in the IC_50_ values could be attributed to differences in the species of bamboo, solvent, fraction method, etc. The lower IC_50_ values of the bamboo stem ethanolic extracts could be linked to the presence of mixtures of phenolic compounds and flavonoids, which donate atoms for scavenging radicals. Although there is a number of studies on polyphenols and flavonoids in bamboo leaves, studies on bamboo stems are rarely reported. Phenolics from the stems of five bamboo species (*Yushania chungii*, *Fargesia robusta*, *Fargesia denudata*, *Fargesia rufa*, and *Fargesia scabrida*) were researched, and the major components were identified as chlorogenic acid, luteolin, and isoorientin [15]. The presence of phytochemicals in the extracts is affected by the solvent used. EtOH possesses both a hydroxyl group and an ethyl group, so the solvent can dissolve both polar and non-polar molecules [16]. However, if the bamboo stem is dried, water can improve the extraction of phytochemicals. Therefore, the most suitable solvent for extracting flavonoids is 80% EtOH.

Bamboo (*P. nigra* var. henonis) shavings, which are the outer culm of the bamboo, are also known to contain polyphenols, mainly chlorogenic acid and *p*-coumaric acid [17]. The outer culm and inner culm of bamboo stems are the most important sources of active compounds, i.e., 8-*C*-glucosyl apigenin, luteolin derivatives, and chlorogenic acid [10]. There could be possible changes in the concentrations or types of compounds depending on the generation and locations. Some researchers have reported changes in phenolic compounds in other bamboo species during ontogeny [18,19] and in response to environmental factors [20,21]. Catechin has many derivatives, such as epicatechin, epigallocatechin, and gallocatechin; therefore, many plant extracts show variable MS results. The catechin MS peak appears at *m*/*z* 289–305 [22]. Chlorogenic acid (3-*O*-caffeoylquinic acid) appears at *m*/*z* 354.095 in MS data. 4-*O*-caffeoylquinic acid and 5-*O*-caffeoylquinic acid, which are isomers of chlorogenic acid, also have an *m*/*z* of 354 [23]. Peaks three, four, and five showed *m*/*z* values of 180.042 (caffeic acid), 164.047 (*p*-coumaric acid), 194.057 (ferulic acid), and these results match the compounds’ molecular weights [24]. In this study, the rutin peak appeared at *m*/*z* 610.153; other researchers also observed the *m*/*z* value of rutin at 610 [25]. However, other researchers found that the *m*/*z* value 611.1612, with a main fragment at 465.1025, was also rutin [26]. Luteolin also has various derivatives and shares the same *m*/*z* value (*m*/*z* 286). When vulnerable epidermal melanocytes are exposed to ultraviolet (UV) rays, melanin synthesis is promoted as a result of ROS overproduction. ROS in the mitochondria and peroxisomes are produced during normal metabolic processes. However, the homeostasis of melanocytes can be disrupted by oxidative stress, compromising their survival or leading to their malignant transformation [27].

There are two protective roles of melanin, which are to reduce the ROS or to inhibit ROS production in melanocytes. To reduce ROS in melanocytes, many antioxidants and antioxidative compounds from plants were evaluated for their antioxidant activities. In this study, we assessed the inhibitory effect of PN3 on DNA nicking by hydroxyl radicals. When plasmid DNA was dissolved in Fenton’s reagent, it showed the formation of single-stranded relaxed nicked DNA (R-form), double-stranded nicked, and linear DNA (L-form). This indicated that PN3 could inhibit DNA damage by Fenton’s reagent. ROS are generated by several different factors, such as hydrogen peroxide (H2O2), UV light, and unstable chemicals. To study ROS development under α-MSH treatment, the ROS-sensitive dye 2′,7′-dichlorodihydrofluorescein diacetate (DCFH-DA) was used for detection. The relative DCF fluorescence intensity was double that of normal cells when the cells were treated with α-MSH. This indicates that α-MSH treatment induces the generation of ROS in B16F10 cells. By contrast, treatment with 50 μg/mL and 100 μg/mL PN3 showed significantly decreased fluorescence intensities. Fenton’s reagent and pUC19 plasmid were used as the ROS and melanocyte DNA alternative, respectively. As a result, PN3 at 100 μg/mL had the most effective inhibition of Fe^3+^-dependent DNA nicking by hydroxyl radicals. The hydroalcoholic extract of *Desmostachya bipinnata* showed DNA damage protection activity both in vitro and in vivo [28]. In the experiments using pUC19, a dose of PN3 10 times higher than that used on the subsequent staining assay was used because the experimental principle is different. In particular, when implementing a Fenton reaction, three concentrations of Fenton reagent, plasmid, and chemical must be simultaneously considered. In the presence of a DNA-damaging agent (Fenton’s reagent), the plant extract protected the DNA from oxidative damage at 50 μg/mL. Epigallocatechin gallate (EGCG), one of the catechins, is a potent ROS scavenger with a strong antioxidant activity [29].

We also observed the generation of ROS in B16F10 melanocytes after treatment with α-MSH. ROS generation was reported in the case of α-MSH-induced cells. Haycock et al. (2000) demonstrated that α-MSH reduces the impact of proinflammatory cytokine and peroxide-generated oxidative stress on keratinocyte and melanoma cell lines [30]. The melanocytes, which are uniquely poised to accomplish their primary function of delivering melanin to the keratinocytes, are under continuous low-grade oxidative insult. Within the melanocytes, the synthesis of their sine qua non-product (melanin) results in the generation of hydrogen peroxide. If inappropriately processed within the melanosome, this molecule can lead to the generation of hydroxyl radicals and other ROS [31]. When we treated PN3 with α-MSH-induced cells, the ROS generation was decreased. DCFH-DA which is very sensitive to ROS, is considered as a useful tool for the quantitative measurement of ROS in living cells [32]. Human skin cells naturally generate melanin pigments as a photoprotection reaction. There are many signaling pathways to produce melanin, and all signals eventually upregulate the microphthalmia-associated transcription factor (MITF). As activated MITF promotes tyrosinase, TRP-1, and TRP-2 expression, melanin is formed. The aim of the present study was to clarify the anti-melanogenic effect of bamboo stem extracts. In this study, PN3 downregulated the expression of tyrosinase, TRP-1, TRP-2, and MITF. Catethins, the main components of PN3, have been proven to inhibit melanin synthesis by downregulating the expression of tyrosinase in B16 melanoma cells [33]. Catechins suppress MITF production and act as a tyrosinase inhibitor during melanogenesis in Mel-Ab melanocytes [33]. Tyrosinase, TRP-1, and TRP-2 are key enzymes involved in melanin biosynthesis. TYR catalyzes two distinct reactions: the conversion of tyrosine to l-DOPA, and of DOPA to dopaquinone. Dopaquinone is spontaneously converted to dopachrome. TRP-2 catalyzes the conversion of dopachrome to 5,6-dihydroxyindole-2-carboxylic acid (DHICA), whereas TRP induces the oxidation of DHICA to indole-5,6-quinone-2-carboxylic acid. As shown in Figure 6, after treatment with PN3 at concentrations of 25, 50, and 100 μg/mL, TYR expression in B16 melanoma cells was significantly reduced by 46.64%, 57.25%, and 78.85%, respectively, in a dose-dependent manner, and the expression levels of TRP-1 were decreased by 56.90%, 59.35%, and 86.99%, respectively. Compared to the control, the effect of 20 μg/mL PN3 was not significantly different in TRP-1 (*p* < 0.05), and in MITF, and that of 100 μg/mL PN3 was significantly lower. MITF regulates the expression of MC1R, and α-MSH binds to its specific MC1R, which enhances MITF expression. The expression of MC1R increases the levels of cAMP; increases in cAMP level induce MITF expression. MITF is a pivotal regulator involved in the expression of melanogenic proteins. As shown in Figure 6, the expression level of MITF was reduced by PN3 in a dose-dependent manner. Compared to the control, the expression levels of MITF were reduced by 89.36% and 99.08% at 25 and 100 μg/mL of PN3, respectively. PN3 induced a significant suppression in mRNA expression levels of *TYR* and *TRP*-1 via *MITF* downregulation, which was caused by reduced intracellular cAMP levels. These results revealed that the inhibitory mechanism of PN3 on melanogenesis involves the cAMP–PKA, and CREB–MITF–TYR pathways. An et al. reported that p-coumaric acid treatment inhibited tyrosinase, TRP-1, and MITF expression [7]. As mentioned before, bamboo stems include various polyphenol compounds, such as catechin and *p*-coumaric acid, and they show very efficient anti-melanogenesis activity. Although this study clearly demonstrated the anti-melanogenic effect of a bamboo stem extract (PN3) in B16F10 melanoma cells, important questions remain as to its in vivo efficacy. In this study, we did use B16F10 cells instead of human normal skin cells (melanocyte) that produce melanin biopolymers because these cells have many common characteristics of normal melanocytes. Some markers of melanocyte differentiation and branch-like dendrite structure formation are easily quantifiable in B16F10 cells [34,35,36]. However, primary melanocytes as well as transformed cell lines would be used to make our argument stronger. Until now, many other tyrosinase inhibitors found in in vitro studies failed to show in vivo efficacy, likely because they could not reach or enter the cells because of the corneum barrier. Therefore, further in vivo studies and clinical trials of bamboo stem extracts (e.g., PN3) are needed to prove their anti-melanogenesis effects.

## 4. Materials and Methods

### 4.1. Chemicals

Ascorbic acid, 2,2-diphenyl-1-picrylhydrazyl (DPPH), Tween-20, thiazolyl blue tetrazolium bromide (MTT), 2,2′-azino-bis(3-ethylbenzthiazoline-6-sulfonic acid) (ABTS), FeSO_4_, salicylic acid, H_2_O_2_ (for hydroxyl radical scavenging), Folin-Ciocalteu reagent, sodium carbonate (for total phenolics), aluminum chloride, potassium acetate (for total flavonoids), quercetin, and mushroom tyrosinase were obtained from Sigma Aldrich (St. Louis, MO, USA). All other chemicals and reagents were of high-quality grade and were commercially available. Antibodies against tyrosinase, TRP-1, TRP-2, MITF, PKA, and CREB were obtained from Santa Cruz Biotechnology (Santa Cruz, CA, USA). Phospho-PKA and phospho-CREB antibodies were provided by Cell Signaling (Danvers, MA, USA). α-Melanocyte-stimulating hormone (α-MSH), dimethyl sulfoxide (DMSO), sodium nitrite, and an antibody against β-actin were obtained from Sigma Aldrich. Horseradish peroxidase-conjugated goat anti-rabbit and anti-mouse antibodies were purchased from Invitrogen (Carlsbad, CA, USA).

### 4.2. Preparation of Bamboo Stem Extracts

Bamboo stems of *Phyllostachys nigra* var. henonis (PN) located in the southern part of Korea were collected from a local supplier in Damyang City, Korea, during the summer season of 2016. The fresh bamboo stems were dried at 60 °C and cut into small pieces ranging in size from 1.0–2.0 cm. The dried stems (10 g) were powdered and extracted with several solvents by the methods described in Figure 1. Powdered bamboo stems were extracted with water at 1.5 atm and 121 °C for 90 min (autoclaving). Various concentration of EtOH (50%, 80%, and 100%) were used to extract bamboo stems for 3–4 days at room temperature (25 ± 2 °C). Boiled water was also used to prepare the extract at 100 ± 1 °C for 24 h (water heating). The five extracts mentioned above were vacuum-concentrated, resuspended with 50% MeOH, and partitioned with n-hexane and EtOAc. The final EtOAc layers from each solvent and method were named as autoclaving (PN1), 50% EtOH (PN2), 80% EtOH (PN3), 100% EtOH (PN4), and boiling water (PN5).

### 4.3. Antioxidative Activity Assay

#### 4.3.1. DPPH Radical Scavenging Activity

The radical scavenging activity was determined using a 2,2-diphenyl-1-picrylhydrazyl (DPPH) radical scavenging assay with some modifications [37,38]. Each extract of 200 μL was mixed with 800 μL of 1 mmol/L methanolic DPPH. The mixtures were left for 15 min in the dark. Then, the absorbance was measured at 517 nm with a SCINCO UV-Vis spectrophotometer S-3100 (Seoul, Korea). The scavenging activity of the DPPH radical was calculated using the following equation: Scavenging activity (%) = 100 × (A0 − A1)/A0, where A0 is the absorbance of the MeOH control, and A1 is the absorbance in the presence of the bamboo stem extracts. The inhibition concentration (IC_50_) was defined as the amount of extract required for a 50% reduction of the initial free radicals produced from DPPH. The IC_50_ values were obtained from the resulting inhibition curves. The results were compared with the activity of ascorbic acid (Sigma Aldrich) as the control.

#### 4.3.2. ABTS Radical Scavenging Activity

2,2′-Azino-bis(3-ethylbenzothiazoline-6-sulfonic acid) (ABTS) was dissolved in water to a 7 mM concentration. The ABTS radical cation was produced by reacting the ABTS stock solution with 2.45 mM potassium persulfate (final concentration) and allowing the mixture to stand in the dark at room temperature for 12–16 h before use [39]. Each extract of 200 μL was mixed with 1000 μL of ABTS solution. The mixtures were left for 15 min in the dark. Then, absorbance was measured at 730 nm with a SCINCO UV-Vis spectrophotometer S-3100 (Seoul, Korea). The scavenging activity of the ABTS radical was calculated using the following equation: Scavenging activity (%) = 100 × (A0 − A1)/A0, where A0 is the absorbance of the water control, and A1 is the absorbance in the presence of the bamboo stem extracts. The inhibition concentration (IC_50_) was defined as the amount of extract required for 50% reduction of free radical scavenging activity. The IC_50_ values were obtained from the resulting inhibition curves. The results were compared with the activity of ascorbic acid (Sigma Aldrich) as a control.

#### 4.3.3. Hydroxyl Radical Scavenging Activity

The hydroxyl radical is one of the potent reactive oxygen species in biological systems. It reacts with the polyunsaturated fatty acid moieties of the cell membrane phospholipids and causes damage to the cells [40]. An aliquot of the extract (0.5 mL) was mixed with 1 mL of 9 mM FeSO4, 1 mL of 9 mM salicylic acid, and 0.5 mL of 0.3% H_2_O_2_. After incubation at room temperature for 30 min, the absorbance of the reaction mixture was measured at 510 nm with a SCINCO UV-Vis spectrophotometer S-3100 (Seoul, Korea). The scavenging activity of the hydroxyl radical was calculated using the following equation: Scavenging activity (%) = 100 × (A0 − A1)/A0, where A0 is the absorbance of the water control, and A1 is the absorbance in the presence of the bamboo stem extracts. The inhibition concentration (IC_50_) was defined as the amount of extract required for a 50% reduction of free radical scavenging activity. The IC_50_ values were obtained from the resulting inhibition curves. The results were compared with the activity of ascorbic acid (Sigma Aldrich) used as a control.

### 4.4. Total Phenol and Flavonoid Contents

The total phenol content in the extracts was determined with the modified Folin-Ciocalteu method [41]. An aliquot of the extract (0.5 mL) was mixed with 0.5 mL of Folin-Ciocalteu reagent and 0.5 mL of 2% sodium carbonate (*w*/*v*). After incubation at room temperature for 30 min, the absorbance of the reaction mixture was measured at 750 nm with a SCINCO UV-Vis spectrophotometer S-3100 (Seoul, Korea). The extract samples were evaluated at a final concentration of 1 mg/mL. The total phenolic content was expressed as mg/mL of p-coumaric acid equivalent using the following equation based on the calibration curve: *y* = 2.9533*x* + 0.1146, R^2^ = 0.9883, where *x* was the absorbance and y was the *p*-coumaric acid equivalent (μg/g).

The total flavonoid content in the extracts was determined by the modified Chang method [42]. The concentrated extract was prepared at a concentration of 5000 ppm using MeOH. An aliquot of each extract (5000 ppm, 0.5 mL) was mixed with 1.5 mL of MeOH, 0.1 mL of 10% aluminum chloride, 0.1 mL of 1 M potassium acetate, and 2.8 mL of distilled water. After incubation at room temperature for 40 min, the absorbance of the reaction mixture was measured at 415 nm with a SCINCO UV-Vis spectrophotometer S-3100 (Seoul, Korea). Quercetin was used to determine the calibration curve, and concentrations ranged from 12.5–120 ppm. The total flavonoid content was expressed as mg/g quercetin equivalent using the following equation based on the calibration curve: *y* = 0.0071*x* + 0.0618, R^2^ = 0.99, where *x* was the absorbance and y was the quercetin equivalent (mg/g).

### 4.5. High-Performance Liquid Chromatography with Diode-Array Detection (HPLC–DAD) Analysis

The most effective extract, PN3, was analyzed quantitatively by high-performance liquid chromatography (HPLC). The Shimadzu HPLC system (Japan) consisted of a LC-20AD pump, a diode array detector (SPD-20A), and a Shim-Pack GIS-ODS C_18_ column (4.6 × 150 mm, 5 μm). The mobile phases for the HPLC consisted of solvent (A), 0.3% (*v*/*v*) acetic acid in water; and solvent (B), 0.3% (*v*/*v*) acetic acid in MeOH. The gradient elution program was as follows: 0–80 min, 10%–60% B. The flow rate was 0.7 mL/min. The column temperature was 40 °C, and the absorption was measured at 254 nm. Samples consisting of 30 μL were injected sequentially. The compounds were identified by comparing the retention times of standard materials.

### 4.6. LC–Q–TOF-MS Analysis

The quantitative analysis of the PN3 extract was performed using a LC-Q–TOF-MS system (G6550A, Agilent Technologies, Santa Clara, CA, USA) equipped with a jet stream electrospray ionization (ESI) interface. The TOF-MS system was operated in positive mode and the mass analysis conditions were set as follows: drying gas (N2) flow rate, 10 L/min; drying gas temperature, 350 °C; fragmentation voltage, 140 V; nebulizer, 45 psi; capillary voltage, 4000 V; skimmer voltage, 65 V; sheath gas temperature, 250 °C; nozzle voltage, 500 V; and octopole RF voltage, 750 V. The mass spectra were acquired in the *m*/*z* range of 100–800. The MS data were collected in the MS scan mode. The molecular masses of the precursor ions were accurately detected using two reference masses (112.9856 and 966.0007). During the analysis, the reference masses were infused to calibrate the MS system. All the operations and the analyses of the data were controlled using MassHunter B.04.00 software (Agilent Technologies).

### 4.7. Tyrosinase Inhibition Assay—A Cell-Free System

The assay was performed according to the protocol described by Macrini et al., 2009, with some modifications [43]. Aliquots of 10 μL of a solution composed of 1250 U/mL of mushroom tyrosinase (Sigma Aldrich) were added to 96-well microplates, and then 70 μL of pH 6.8 phosphate buffer solution and 60 μL of PN3 (350 μg/mL, in 25% DMSO) was also added. For the positive control, 60 μL of kojic acid (17.5 μg/mL in 25% DMSO) was used, and, for the negative control, 60 μL of 2.5% DMSO was added. To the resulting mixture, 70 μL of l-tyrosine (Sigma Aldrich) was added at a concentration of 0.3 mg/mL in distilled water (the final volume in the wells was 210 μL). The absorbance of the microplate wells was read using SpectraCount microplate spectrophotometer (Packard, Conroe, TX, USA) at 510 nm (T0). Then, the microplates were incubated at 30 ± 1 °C for 60 min and the absorbance was measured again (T1). An additional incubation period of 60 min at 30 ± 1 °C was performed, after which a new spectrophotometric reading was taken (T2). The inhibitory percentages at the two time points (T1 and T2) were obtained according to the formula: 𝐼𝐼𝐼𝐼% = 𝑐𝑐 − 𝑆𝑆 × 𝑐𝑐 100, where IA% = inhibitory activity; C = negative control absorbance; S = sample or positive control absorbance (absorbance at time T1 or T2 minus the absorbance at time T0) [44].

### 4.8. Cell Culture

B16F10 melanoma cells, a murine melanoma cell line, were purchased from ATCC (American Type Culture Collection; Rockville, MD, USA). The cells were maintained in Dulbecco’s modified Eagle’s medium (DMEM) supplemented with 10% fetal bovine serum (FBS), 50 units/mL penicillin, and 50 μg/mL streptomycin at 37 °C in a humidified atmosphere with 5% CO_2_ at 37 °C.

### 4.9. MTT Cell Viability Assay

The cytotoxicity proliferation assay was carried out using 3-(4,5-dimethylthiazol-2-yl)-2,5-diphenyltetrazolium bromide (MTT). B16F10 cells were cultured at 1 × 104 cells/cm^3^ in 24-well plates. After 24 h, the cells were treated with PN3 (25, 50, and 100 μg/mL) for 48 h. At the end of the incubation, 100 μL of MTT solution (1 mg/mL in DMEM) was added to each well. After incubation at 37 °C for 1 h, the medium was gently removed, and 400 μL of dimethyl sulfoxide (DMSO) was added. The absorbance of each well was measured at 570 nm using a spectrophotometer.

### 4.10. Measurement of Melanin Content

The melanin content was determined according to the modified methods of Hosoi et al., 1985 [45]. B16F10 cells were cultured at 1 × 10^4^ cells/cm in 12-well plates. After 24 h, the cells were stimulated with α-MSH 1 μg/mL. At the same time, various concentrations of PN3 (25, 50, and 100 μg/mL) were added for 48 h. After washing with phosphate-buffered saline (PBS), the cells were harvested by trypsinization. The cell pellet was solubilized in 200 μL of 1 N NaOH. The absorbance of each well was measured at 405 nm using a spectrophotometer.

### 4.11. Immunoblot Analysis

For protein extraction and subcellular fractionation, SDS-polyacrylamide gel electrophoresis and immunoblot analyses were performed according to previously published procedures [46]. Briefly, the samples were separated by 7.5% gel electrophoresis and were electrophoretically transferred to a nitrocellulose paper. The nitrocellulose paper was incubated with the indicated primary antibodies, followed by incubation with horseradish peroxidase-conjugated secondary antibodies. The immunoreactive proteins was visualized by enhanced chemiluminescence (ECL) detection (Amersham Biosciences, Buckinghamshire, UK). The equal loading of proteins was verified by β-actin immunoblotting.

### 4.12. Fenton Reaction Inhibition Assay

Fenton reaction assays of PN3 at different concentrations were carried out. Amounts of PN3 at 250–1000 μg/mL were added to prevent the oxidation of pUC19 plasmid by the Fenton’s reagents. DCFH-DA is a cell-permeable, non-fluorescent probe that is cleaved by intracellular esterases and turns into highly fluorescent dichlorofluorescein upon reaction with H_2_O_2_. After α-MSH 1 μM treatment of B16F10 cells for 3 h, the cells were stained with 10 μM DCFH-DA at 37 °C for 30 min. H_2_O_2_ generation was determined by measuring dichlorofluorescein using a fluorescence microplate reader (Jemini, Molecular Device, Sunnyvale, CA, USA) at excitation and emission wavelengths of 485 and 530 nm.

### 4.13. RNA Isolation and RT-PCR

Total RNA was extracted using Trizol (Invitrogen, Carlsbad, CA, USA) in accordance with the manufacturer’s instructions. To obtain cDNA, the total RNA (2 μg) was reverse transcribed using an oligo (dT) 16 primer. The cDNA was amplified using a high-capacity cDNA synthesis kit (Bioneer, Daejon, Korea) with a thermal cycler (BioRad, Hercules, CA, USA). Subsequently, PCR was performed using a PCR premix (Bioneer, Daejon, Korea), and real-time RT-PCR was performed with STEP ONE (Applied Biosystems, Foster City, CA, USA) using a SYBR Green premix in accordance with the manufacturer’s instructions (Applied Biosystems). Primers were synthesized by Bioneer. The following primer sequences were used: mouse *TYR*, 5′-ATAACAGCTCCCACCAGTGC-3′ (sense) and 5′-CCCAGAAGCCAATGCACCTA-3′ (antisense); mouse *MITF*, 5′-CTGTACTCTGAGCAGCAGGTG-3′ (sense) and 5′-CCCGTCTCTGGAAACTTGATCG-3′ (antisense); mouse *TRP*-1, 5′-AGACGCTGCACTGCTGGTCAAGCCTGTAGCCCACGTCGTA-3′ (sense) and 5′-GCTGCAGGAGCCTTCTTTCT-3′ (antisense). The expression of glyceraldehyde 3-phosphate dehydrogenase (*GAPDH*) was used as an endogenous control for the quantitative RT-PCR experiments.

### 4.14. Statistical Analysis

For the statistical analysis of the data, IBM SPSS online version 23.0 (SPSS, Inc., Chicago, IL, USA, an IBM company) was used. One-way analysis of variance (ANOVA) was used to assess the statistical significance of the differences among treatment groups. For each statistically significant effect of treatment, the Duncan’s multiple range test was used for comparisons between multiple group means. The data were expressed as means ± standard deviation (S.D.).

## 5. Conclusions

This study demonstrated the antioxidative and anti-melanogenic activities of Korean bamboo (*Phyllostachys nigra* var. henosis) stem extracts (especially the EtOAc fraction) in a cell-free system and B16F10 melanoma cells. The fraction consisted in a mixture of polyphenols and flavonoids, such as catechin, chlorogenic acid, caffeic acid, and *p*-coumaric acid. The anti-melanogenic activity was thought to depend on PKA/CREB-mediated MITF downreguration, resulting in the suppression of melanin production. The bamboo stem would be a valuable asset as an additive to whitening cosmetics once the fractions are standardized, and an appropriate clinical trial is done.

## Figures and Tables

**Table 1:** IC_50_ values of the radical scavenging activity of bamboo stem extracts.

Bamboo Sample	DPPH	ABTS	Hydroxyl Radical
PN1	1064.48 ± 107.77 ^e^	704.99 ± 28.53 ^c^	698.68 ± 24.40 ^c,d^
PN2	714.35 ± 39.54 ^c^	463.81 ± 13.93 ^b^	560.19 ± 21.85 ^b,c^
PN3	565.63 ± 17.75 ^b^	414.61 ± 35.12 ^b^	509.17 ± 33.76 ^b^
PN4	2019.67 ± 69.40 ^f^	669.68 ± 35.62 ^c^	766.73 ± 22.23 ^d^
PN5	877.34 ± 63.75 ^d^	2258.18 ± 125.06 ^d^	1395.93 ± 222.72 ^e^
Positive control	23.02 ± 0.39 ^a^	51.86 ± 0.72 ^a^	90.57 ± 0.60 ^a^

PN1, EtOAc fraction of autoclaved extract; PN2, EtOAc fraction of 50% EtOH extract; PN3, EtOAc fraction of 80% EtOH extract; PN4 EtOAc fraction of 100% EtOH extract; PN5, EtOAc fraction of hot water extract; positive control, ascorbic acid; DPPH, 2,2-diphenyl-1-picrylhydrazine; ABTS, 2,2′-azino-bis(3-ethylbenzothiazoline-6-sulfonic acid). Each piece of data is the mean ± standard deviation (SD) of three experiments; ^a–f^ values in the same column not sharing a common superscript are significantly different by Duncan’s multiple range test (*p* < 0.05).

**Table 2:** Total polyphenol and total flavonoid contents of bamboo stem extracts.

Bamboo Sample	Total Polyphenols (COAE mg/g)	Total Flavonoids (QE mg/g)
PN1	0.140 ± 0.015 ^a^	8.09 ± 0.22 ^b^
PN2	0.148 ± 0.014 ^a^	9.24 ± 0.70 ^c,d^
PN3	0.179 ± 0.022 ^b^	9.57 ± 0.37 ^d^
PN4	0.130 ± 0.017 ^a^	8.64 ± 0.01 ^b,c^
PN5	0.131 ± 0.016 ^a^	7.35 ± 0.27 ^a^

COAE, *p*-coumaric acid equivalent; QE, quercetin equivalent; ^a–d^ values in the same column not sharing a common superscript are significantly different by Duncan’s multiple range test (*p* < 0.05).

## Supplementary Materials

Supplementary materials can be found at http://www.mdpi.com/1422-0067/19/2/409/s1.

## Abbreviations

ABTS	2,2′-azino-bis(3-ethylbenzothiazoline-6-sulfonic acid)
AKT	protein kinase B
α-MSH	α-Melanocyte-stimulating hormone
cAMP	cyclic adenosine monophosphate
COA	*p*-coumaric acid
COAE	*p*-coumaric acid equivalent
CREB	cAMP response element-binding protein
DCFH-DA	dichloro-dihydro-fluorescein diacetate
DHICA	5,6-dihydroxyindole-2-carboxylic acid
DMSO	dimethyl sulfoxide
DOPA	3,4-dihydroxyphenylalanine
DPPH	2,2-diphenyl-1-picrylhydrazyl
EtOAc	ethyl acetate
EtOH	ethanol
GSK-3	glycogen synthase kinase 3
HPLC	high-performance liquid chromatography
IC_50_	inhibitory concentration 50
LC–Q–TOF–MS	liquid chromatography–quadropole–time of flight-mass spectrometry
MAPKs	mitogen-activated protein kinase
MC1R	melanocortin 1 receptor
MeOH	methanol
MITF	microphthalmia-associated transcription factor
MS	mass spectrometry
MTT	3-(4,5-dimethylthiazol-2-yl)-2,5-diphenyltetrazolium bromide
ORAC	oxygen radical absorbance capacity
p-CREB	phosphorylation of cAMP response element-binding protein
p-PKA	phosphorylation of protein kinase A
PKA	protein kinase A
PI3K	phosphatidylinositol-4,5-bisphosphate 3-kinase
PN3	ethylacetate fraction of 80% ethanol extracts from the leaves of *Phyllostachys nigra* var. henonis
Q	quercetin
QE	quercetin equivalent
ROS	reactive oxygen species
RT	retention time; bromide
RT-PCR	reverse transcription polymerase chain reaction
SOD	superoxide dismutase
TE	trolox equivalent
TRP-1	Tyrosinase-related protein 1
TRP-2	Tyrosinase-related protein 2
TYR	Tyrosinase
