# Supplementary material for: Antioxidative and Anti-Melanogenic Activities of Bamboo Stems (Phyllostachys nigra variety henosis) via PKA/CREB-Mediated MITF Downregulation in B16F10 Melanoma Cells
Source: Int J Mol Sci. 2018 Jan 30;19(2):409. doi: 10.3390/ijms19020409 (PMC5855631; doi:10.3390/ijms19020409)
Supplement: Supplementary file 1 [file ijms-19-00409-s001.pdf]

Table S1. Linear regression equation of radical scavenging assay results

| | DPPH | ABTS | Hydroxyl radical |
|---|---|---|---|
| PN1 | y = 0.0119x + 37.299 | y = 0.0305x + 28.445 | y = 0.8485x + 10.527 |
| PN2 | y = 0.0248x + 32.268 | y = 0.0284x + 36.827 | y = 0.0334x + 26.633 |
| PN3 | y = 0.0265x + 35.005 | y = 0.0291x + 37.934 | y = 0.0291x + 33.675 |
| PN4 | y = 0.0102x + 29.424 | y = 0.0316x + 28.804 | y = 0.029x + 35.211 |
| PN5 | y = 0.0207x + 31.823 | y = 0.0067x + 35.011 | y = 0.0335x + 24.309 |
| Positive control | y = 0.6159x + 35.822 | y = 0.9846x - 1.0621 | y = 0.0156x + 26.222 |

PN1, EtOAc fraction of autoclaving extract; PN2, EtOAc fraction of 50% EtOH extract; PN3, EtOAc fraction of 80% EtOH extract; PN4 EtOAc fraction of 100% EtOH extract; PN5, EtOAc fraction of hot water extract; Positive control, ascorbic acid

Data is mean±standard deviation (SD) of three times experiments.

A)

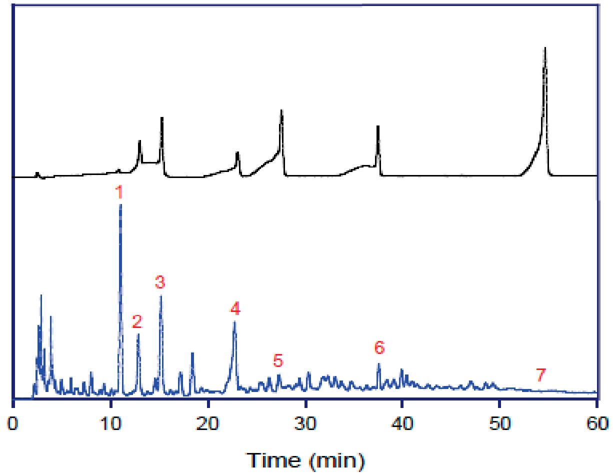


B)

| No. | Compounds | PN3 | |
|---|---|---|---|
| | | R. time (min) | Content (mg/g) |
| 1 | Catechin | 11.05 | 701.39 |
| 2 | Chlorogenic acid | 12.90 | 297.65 |
| 3 | Caffeic acid | 15.21 | 241.94 |
| 4 | p-Coumaric acid | 22.77 | 193.11 |
| 5 | Ferulic acid | 27.31 | 18.14 |
| 6 | Rutin | 37.55 | 66.85 |
| 7 | Luteolin | 54.53 | 3.15 |

C)

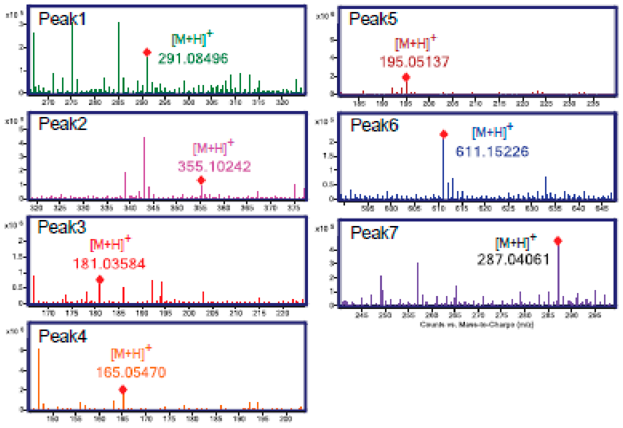


D)

| No. | RT (min) | $[M+H]^+$ | Calculated *m/z* | Formula | Tentative identification |
|---|---|---|---|---|---|
| 1 | 5.102 | 291.086 | 290.079 | $C_{15}H_{14}O_6$ | Catechin |
| 2 | 4.935 | 355.102 | 354.095 | $C_{16}H_{18}O_9$ | Chlorogenic acid |
| 3 | 5.480 | 181.049 | 180.042 | $C_9H_8O_4$ | Caffeic acid |
| 4 | 7.323 | 165.054 | 164.047 | $C_9H_8O_3$ | p-Coumaric acid |
| 5 | 8.346 | 195.065 | 194.057 | $C_{10}H_{10}O_4$ | Ferulic acid |
| 6 | 9.324 | 611.160 | 610.153 | $C_{27}H_{30}O_{16}$ | Rutin |
| 7 | 12.406 | 287.055 | 286.047 | $C_{15}H_{10}O_6$ | Luteolin |

Figure S1. (A) High-performance liquid chromatography (HPLC) chromatograms, (B) contents of the seven compounds, (C) liquid chromatography coupled with quadrupole time-of-flight mass spectrometry (LC-Q-TOF-MS) chromatograms, (D) and mass spectrometry (MS) data of the seven compounds in PN3. Unnumbered peaks of (A) were unknown.
